# Structural and Immunochemical Studies of the Lipopolysaccharide from the Fish Pathogen, *Aeromonas bestiarum* Strain K296, Serotype O18

**DOI:** 10.3390/md11041235

**Published:** 2013-04-17

**Authors:** Anna Turska-Szewczuk, Buko Lindner, Iwona Komaniecka, Alicja Kozinska, Agnieszka Pekala, Adam Choma, Otto Holst

**Affiliations:** 1 Department of Genetics and Microbiology, Maria Curie-Sklodowska University, Akademicka 19, Lublin 20-033, Poland; E-Mails: ikoma@hektor.umcs.lublin.pl (I.K.); adam.choma@poczta.umcs.lublin.pl (A.C.); 2 Division of Immunochemistry, Research Center Borstel, Leibniz-Center for Medicine and Biosciences, Parkallee 10, D-23845 Borstel, Germany; E-Mail: blindner@fz-borstel.de; 3 Department of Fish Diseases, National Veterinary Research Institute, Partyzantow 57, Pulawy 24-100, Poland; E-Mails: koala@piwet.pulawy.pl (A.K.); a.pekala@piwet.pulawy.pl (A.P.); 4 Division of Structural Biochemistry, Research Center Borstel, Leibniz-Center for Medicine and Biosciences, Airway Research Center North (ARCN), Member of the German Center for Lung Research (DZL), Parkallee 4a/c, D-23845 Borstel, Germany; E-Mail: oholst@fz-borstel.de

**Keywords:** lipopolysaccharide, LPS, *O*-specific polysaccharide, OPS, *Aeromonas bestiarum*, fish pathogen, 6dTal

## Abstract

Chemical analyses and mass spectrometry were used to study the structure of the lipopolysaccharide (LPS) isolated from *Aeromonas bestiarum* strain K296, serotype O18. ESI-MS revealed that the most abundant *A. bestiarum* LPS glycoforms have a hexa-acylated or tetra-acylated lipid A with conserved architecture of the backbone, consisting of a 1,4′-bisphosphorylated β-(1→6)-linked d-GlcN disaccharide with an AraN residue as a non-stoichiometric substituent and a core oligosaccharide composed of Kdo_1_Hep_6_Hex_1_HexN_1_P_1_. 1D and 2D NMR spectroscopy revealed that the *O*-specific polysaccharide (OPS) of *A. bestiarum* K296 consists of a branched tetrasaccharide repeating unit containing two 6-deoxy-l-talose (6dTal*p*), one Man*p* and one Gal*p*NAc residues; thus, it is similar to that of the OPS of *A. hydrophila* AH-3 (serotype O34) in both the sugar composition and the glycosylation pattern. Moreover, 3-substituted 6dTal*p* was 2-*O*-acetylated and additional *O*-acetyl groups were identified at O-2 and O-4 (or O-3) positions of the terminal 6dTal*p*. Western blots with polyclonal rabbit sera showed that serotypes O18 and O34 share some epitopes in the LPS. The very weak reaction of the anti-O34 serum with the *O*-deacylated LPS of *A. bestiarum* K296 might have been due to the different *O*-acetylation pattern of the terminal 6dTal*p*. The latter suggestion was further confirmed by NMR.

## 1. Introduction

*Aeromonas* spp. bacteria are ubiquitous in various aquatic ecosystems, such as sea water, freshwater, estuarine and coastal waters, and are even found in chlorinated potable water. They occur commonly in soil habitats and are also frequently isolated from raw and processed food. They are either mesophilic, motile or psychrophilic non-motile Gram-negative rods [1,2,3]. *Aeromonas* bacteria identified as members of the gut microflora in fish and other aquatic animals (amphibians, reptiles) may cause various diseases under environmental stress conditions (overcrowding, poor water quality organic pollution and hypoxia) [4,5]. The species *Aeromonas hydrophila*, *Aeromonas salmonicida*, *Aeromonas caviae*, *Aeromonas veronii*, *Aeromonas sobria* and *Aeromonas bestiarum* have been described as important fish pathogens. They cause chronic disease with skin ulceration or acute systemic infection, referred to as motile aeromonad septicemia (MAS), as well as other pathological lesions [5,6,7]. Contaminated water and food may be a source of human infections. Of the 21 species classified on the basis of DNA-DNA hybridization, *A. hydrophila*, *A. caviae* and *A. veronii* bt. *sobria* are the most common species known to cause human infections. They have been recognized as agents of both intestinal (gastroenteritis, child diarrhea, traveler’s diarrhea, dysentery) and life-threatening extraintestinal infections (septicemia, wound infections, urinary tract infections and, occasionally, meningitidis and peritonitis), especially in immunocompromised patients and children [5,8,9]. 

The pathogenesis of *Aeromonas* infections is a multifactorial complex, as aeromonads produce a wide variety of potential virulence factors, including hemolysins, cytotonic and cytotoxic enterotoxins, proteases, lipases and leukocidins. A type II secretion system (secretion of enterotoxin-Act) and a type III secretion system (T3SS) are crucial in the pathogenesis of *Aeromonas*-associated infections [8]. Moreover, cell-surface components, such as outer membrane proteins, lipopolysaccharide (LPS), the *S*-layer, polar flagella and pili (type IV and bundle-forming pili), have been identified as *Aeromonas* putative virulence factors [10,11,12,13]. The protein *S*-layer that is produced especially by virulent strains of the psychrophilic fish pathogen *A. salmonicida* covers the entire bacterial cell and plays a crucial role in the early stages of infection. The *S*-layer enhances certain physical attributes of the bacterium, including increases in cellular hydrophobicity, cell aggregation and cell-to-tissue adhesion [14]. An equally important non-fimbrial adhesion factor that has been implicated in the pathogenesis of *Aeromonas* spp. is LPS. As an adhesin, *S*-type LPS is indispensable for initial attachment of bacteria to host tissue and necessary during infection events, where it protects bacteria from complement-mediated killing and antimicrobial peptides [5]. It is conceivable that some virulence factors located in the outer membrane or at the bacterial surface require the presence of *O*-antigens for proper expression or functionality. Studies of antigen expression of *A. hydrophila* in biofilms have revealed that a complete LPS structure is important for the ability of *Aeromonas* species to maintain the proper structure of the tetragonal *S*-layer on the cell surface, and the structural changes in the LPS molecule can result in the loss of appropriate conformation of the protein structure [15]. Moreover, the *O*-specific polysaccharide (OPS) was implicated as a factor promoting adhesion of *Aeromonas* bacilli to HEp-2 cells, and switching off of the OPS by using specific monoclonal antibodies significantly reduced bacterial adherence [16,17]. 

Structural analyses of the OPS of several *Aeromonas* strains revealed their heteropolymeric character [18]. The OPS of *A. salmonicida* A449 isolated from salmonids was composed of a Rha-ManNAc unit with one Glc residue and an *O*-acetyl group as substituents [19]. An *A. hydrophila* AH-3 OPS repeating unit was built up of 6-deoxy-l-talose (6dTal), Man and GalNAc residues [20]. Recently, two new structures of *O*-antigens were established for the species *A. bestiarum*, whose strains are frequently isolated in the course of MAS in Polish carp farm [21,22,23]. 

In this work, we report on the chemical structure of the LPS from *A. bestiarum* strain K296 serotype O18, which was isolated from pathologically altered tissue of carp (*Cyprinus carpio* L.) [24]. 

## 2. Results and Discussion

### 2.1. Isolation of LPS and SDS-PAGE

**Figure 1:**
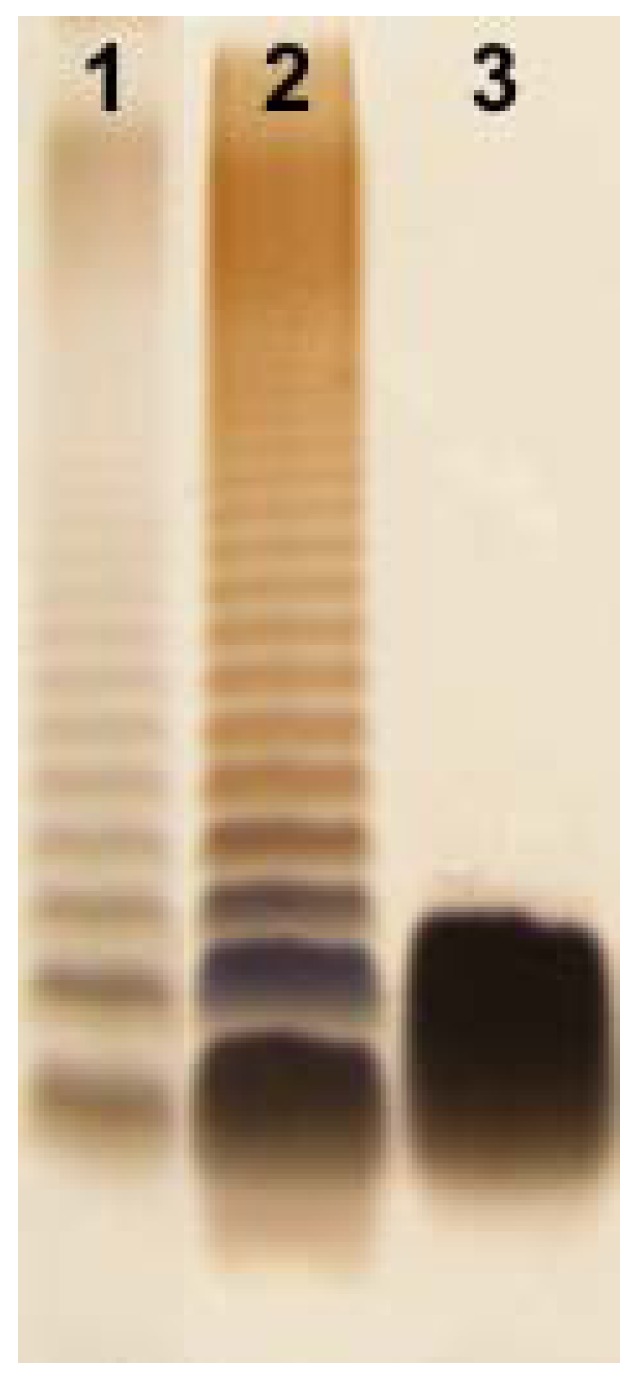
Silver-stained SDS-Tricine PAGE of the *S*- and *R*-type lipopolysaccharide (LPS) of *A. bestiarum* strain K296 (lane 2, 4 μg; lane 3, 4 μg, respectively) and *Salmonella enterica* sv. Typhimurium (Sigma, St. Louis, MO, USA) as reference (lane 1, 2 μg).

*Aeromonas bestiarum* K296 LPS was isolated by hot phenol-water extraction [25] of enzymatically digested bacterial cells [22]. It was found that the LPS was distributed between the phenol and water phases as hydrophobic and hydrophilic LPS fractions in yields of 1.6% and 2.5% of the dry bacterial cell mass, respectively. The SDS-PAGE analysis (Figure 1) of these preparations revealed that the smooth, *S*-type LPS depicting a typical ladder-like pattern was recovered from the phenol phase and the rough, *R*-type LPS from the water phase. The yield of the LPS extracted from the phenol, which was less than half of the yield obtained from the water indicated a lower content of LPS molecules substituted with OPS in the cell envelope of *A. bestiarum* K296. 

### 2.2. Chemical and ESI FT-ICR Mass Spectrometric Analyses of LPS Preparations

The compositional analyses of the LPS preparations were performed by GC-MS of the alditol acetates derived after full acid hydrolysis. The *R*-type LPS contained Glc, Gal, GlcN, d,d-Hep and l,d-Hep in a molar ratio of approximately 2.1:1:1:2.1:4.2. All these sugars were also found in the *S*-type LPS. Additionally, the chemical analysis confirmed the presence of 6-deoxyhexose (6dHex), Man and GalN in this preparation, in a molar ratio of 2:1:0.9. Kdo (3-deoxy-d-*manno*-oct-2-ulosonic acid)—the only acidic sugar—was found in both the *S*- and *R*-type LPS preparations. GC-MS analysis of the fatty acids as methyl esters and *O*-TMS derivatives, obtained after isolation and methanolysis of lipid A, showed the presence of 3-hydroxy myristate [14:0(3-OH)] and dodecanoic (12:0) fatty acids as the most abundant species. GlcN was identified as the sugar component of the lipid A. 

**Figure 2:**
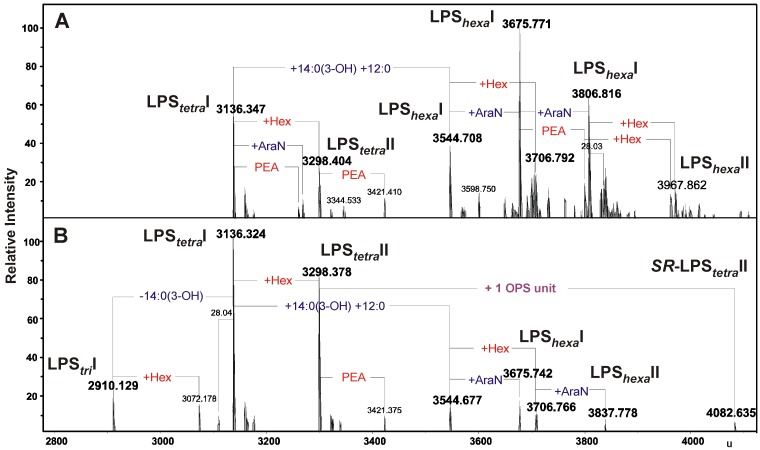
Charge-deconvoluted ESI FT-ICR mass spectra (negative ion mode) of the intact *R*- and *S*-type LPS of *A. bestiarum* strain K296 (**A**) and (**B**), respectively. The composition of major ions is shown close to the corresponding ions. Mass numbers given refer to the monoisotopic masses. LPS*_tetra_*, LPS*_hexa_*, acylation state of the lipid A. LPS*_tetra_*I, tetra-acylated lipid A; LPS*_hexa_*I, hexa-acylated lipid A; *SR*-LPS*_tetra_*II, LPS glycoform II.

The LPS preparations from *A. bestiarum* K296 were analyzed by ESI FT-ICR mass spectrometry. The charge-deconvoluted ESI-MS (negative-ion mode) (Figure 2A,B) of the intact *R*- and *S*-type LPS showed a complex pattern of molecular ion peaks originating from heterogeneity of lipid A, as well as the core oligosaccharide. The heterogeneity was caused by non-stoichiometric substitutions with 4-amino-4-deoxyarabinose (Ara4N, ∆m = 131 u), phosphoethanolamine (PEA, ∆m = 123 u), hexose (Hex, ∆m = 162 u), one or two fatty acid residues, 14:0(3-OH) (∆m = 226 u) and 14:0(3-OH) + 12:0 (∆m = 408.36 u), respectively, and peaks originating from different acyl chain length. The mass spectra of the intact *R*- and *S*-type LPS showed, e.g., two molecular ions at 3136.347 and 3544.708 u, corresponding to the LPS glycoforms with the core nonasaccharide linked to tetra-acylated (LPS*_tetra_*I) and hexa-acylated lipid A (LPS*_hexa_*I), respectively, which had the 1,4′-bisphosphorylated diglucosaminyl backbone (Figure 2, Table 1). Additionally, the mass spectrum of the *S*-type LPS (Figure 2B) showed a molecular ion at 4082.635 u, which, most probably, corresponded to the molecular mass of LPS glycoform II (*SR*-LPS*_tetra_*II) carrying one *O*-antigen repeating unit 6dHex_2_Ac_3_HexNAc_1_Hex_1_-H_2_O (calculated mass 783.2797 u), since the measured mass difference of ∆m = 783.279 u was in excellent agreement with the chemical structure determined by the NMR analysis (see Section 2.3). 

**Figure 3:**
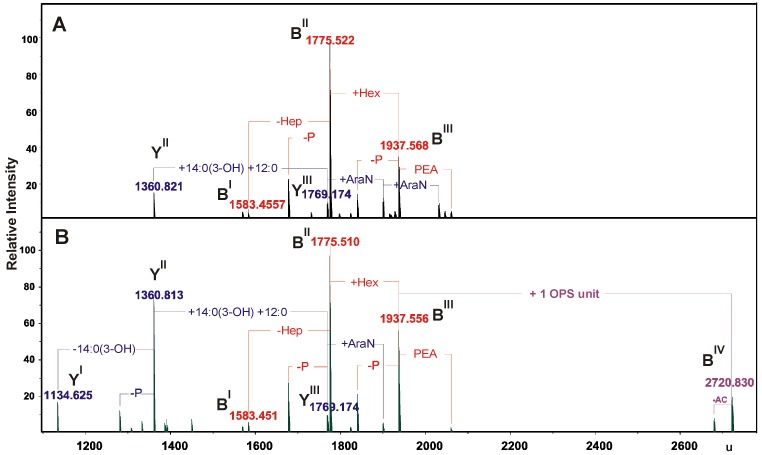
Part of the charge-deconvoluted ESI FT-ICR mass spectra (negative ion mode) of the *R*- and *S*-type LPS under unspecific fragmentation in the collision cell (collision voltage 30 V) (**A**) and (**B**), respectively, providing the fragmentation of the labile linkage between the Kdo and lipid A. Mass numbers given refer to the monoisotopic masses. Ac, an acetyl group.

For a more detailed interpretation, both LPSs were fragmented by unspecific fragmentation in the collision cell, leading to rupture of the labile ketosidic linkage between lipid A and the Kdo of the core oligosaccharide (Figure 3). The mass spectra showed the presence of B fragment ions at 1937.556, 1775.51 and 1583.451 u, originating from the core glycoforms and of Y-ions from the lipid A species [26]. In addition to these ions, a further B ion (2720.830 u) was observed in the spectrum of the *S*-form LPS carrying one OPS unit. Furthermore, the fragment ion spectra have proven that Ara4N was linked in non-stoichiometric amounts to the lipid A species, whereas phosphoethanolamine was linked exclusively to the core oligosaccharides. Based on the chemical component analysis and the accurate mass spectrometric data, the mass spectra were interpreted in detail and the results are summarized in Table 1, Table 2.

**Table 1:** Composition of the main species present in the charge-deconvoluted (negative ion mode) ESI FT-ICR mass spectra of the intact *S-* and *R-*type LPS from *A. bestiarum* strain K296.

Species	M_measured_	M_calculated_	Composition
**LPS*_tri_*I**	2910.129	2910.128	Hep_6_HexHexN_3_KdoP_3_[14:0(3-OH)]_2_12:0
**LPS*_tetra_*I**	3136.324	3136.318	Hep_6_HexHexN_3_KdoP_3_[14:0(3-OH)]_3_12:0
	3136.347	3136.318	
**LPS*_tetra_*II**	3298.378	3298.370	Hep_6_Hex_2_HexN_3_KdoP_3_[14:0(3-OH)]_3_12:0
	3298.404	3298.370	
**LPS*_hexa_*I**	3544.677	3544.668	Hep_6_HexHexN_3_KdoP_3_[14:0(3-OH)]_4_12:0_2_
	3544.708	3544.668	
**LPS*_hexa_*I**	3675.742	3675.713	Hep_6_HexHexN_3_PenNKdoP_3_[14:0(3-OH)]_4_12:0_2_
	3675.771	3675.713	
**LPS*_hexa_*II**	3706.766	3706.720	Hep_6_Hex_2_HexN_3_KdoP_3_[14:0(3-OH)]_4_12:0_2_
	3706.792	3706.720	
**LPS*_hexa_*I**	3806.816	3806.758	Hep_6_HexHexN_3_PenN_2_KdoP_3_[14:0(3-OH)]_4_12:0_2_
**LPS*_hexa_*II**	3837.778	3837.765	Hep_6_Hex_2_HexN_3_PenNKdoP_3_[14:0(3-OH)]_4_12:0_2_
**LPS*_hexa_*II**	3967.862	3968.810	Hep_6_Hex_2_HexN_3_PenN_2_KdoP_3_[14:0(3-OH)]_4_12:0_2_
***SR*-LPS*_tetra_*II**	4082.635	4081.648	6dHex_2_Hep_6_Hex_3_HexN_4_KdoP_3_Ac_4_[14:0(3-OH)]_3_12:0

**Table 2:** Composition of the main Y and B ions present in the charge-deconvoluted (negative ion mode) ESI FT-ICR mass spectra of the *S*- and *R*-type LPS from *A. bestiarum* strain K296 obtained by unspecific fragmentation.

Species	M_measured_	M_calculated_	Composition
**Y^I^**	1134.625	1134.621	HexN_2_P_2_[14:0(3-OH)]_2_12:0
**Y^II^**	1360.813	1360.811	HexN_2_P_2_[14:0(3-OH)]_3_12:0
	1360.821		
**B^I^**	1583.451	1583.447	Hep_5_HexHexNKdoP
	1583.455		
**Y^III^**	1769.174	1769.161	HexN_2_P_2_[14:0(3-OH)]_4_(12:0)_2_
**B^II^**	1775.510	1775.507	Hep_6_HexHexNKdoP
	1775.522		
**B^III^**	1937.556	1937.559	Hep_6_Hex_2_HexNKdoP
	1937.568		
**B^IV^**	2720.830	2720.837	6dHex_2_Hep_6_Hex_3_HexN_2_KdoPAc_4_

The composition of the *A. bestiarum* K296 LPS was further supported by studies of the *O*-deacylated *R*-type LPS (data not shown). The ion at 952.464 u corresponded to the molecular mass of *O*-deacylated lipid A containing a 1,4′-bisphosphorylated GlcN′-(1′→6)-GlcN disaccharide and only the 14:0(3-OH) amide-linked fatty acids. The ions derived from the core region were unchanged. 

### 2.3. Structural Studies of the *O*-Deacetylated OPS

The OPS was released from the hydrophobic LPS by mild-acid degradation, followed by gel-permeation chromatography (GPC). Sugar analysis of the OPS using GC-MS of the acetylated alditols identified 6dTal, Man and GalN in a relative peak area ratio of 2:1:0.7. 6dTal was identified by comparing its retention time and mass spectrum to those obtained from the reference compounds isolated from the OPS of *Mesorhizobium huakuii* strain S-52 [27] and *A. hydrophila* strain AH-3 [20]. The absolute configuration of monosaccharides [28] identified the configuration of 6dTal as l and that of the other constituent sugars as d. 

Linkage analysis by GC-MS of the partially methylated alditol acetates derived from the methylated polysaccharide resulted in identification of terminal 6dTal*p*, 3-substituted 6dTal*p*, 3-substituted Gal*p*N and 3,4-disubstituted Man*p*. 

The initial OPS of *A. bestiarum* K296 was *O*-acetylated, as followed from the presence of methyl group signals at δ_H_ 2.15–2.20 and δ_C_ 21.2–21.3 in the ^1^H and ^13^C NMR spectra, respectively. After *O*-deacetylation of the OPS, the ^1^H and ^13^C NMR spectra lost their structural heterogeneity, which, most likely, was caused by non-stoichiometric *O*-acetylation, and showed that the polymer had a regular structure composed of branched tetrasaccharide repeating units. 

The ^13^C NMR spectrum (Figure S1A, see Supplementary Information) of the *O*-deacetylated OPS contained signals for four anomeric carbons at δ 97.6, 98.6, 101.6 and 104.0; a signal for one nitrogen-bearing carbon (GalN C-2) at δ 52.5; two methyl groups of 6-deoxysugars (6dTal) at δ 16.3; *N*-acetyl groups (CH_3_ at δ 23.0 and CO at δ 175.5) and other non-anomeric sugar ring carbons in the region δ 66.3–79.9, some of which overlapped. The ^13^C NMR data showed that all the sugar residues were in the pyranose form [29], as no signals for ring carbons above δ 81, diagnostic of furanose, were detected. 

The ^1^H NMR spectrum of the *O-*deacetylated OPS (Figure S1B) contained signals for four anomeric protons at δ 5.10, 5.06, 5.01 and 4.49, labeled **A** through **D**, respectively. In the high field region of the spectrum, there were also signals originating from the methyl groups of two 6dTal residues at δ 1.24 and 1.29 and one signal of a *N*-acetyl group at δ 2.05. 

The anomeric configuration of each monosaccharide was assigned on the basis of the ^3^*J*_H-1,H-2_ (measured on the DQF-COSY spectrum) and ^1^*J*_C-1,H-1_ coupling constants and the *intra*-residual NOE contacts identified in the NOESY spectrum, whereas the ring configuration of each residue was inferred by the vicinal ^3^*J*_H,H_ coupling constants [29]. Chemical shifts of each spin system were assigned in ^1^H-^1^H, TOCSY, DQF-COSY, NOESY, ^1^H-^13^C HSQC and ^1^H-^13^C HMBC experiments. All chemical shifts are summarized in Table 3. 

Based on these data, the spin systems were assigned to four residues, one Man*p*, two 6dTal*p* and one Gal*p*NAc. In particular, the chemical shifts for H-5 and C-5 at δ_H_ 3.99 and δ_C_ 71.9, respectively, indicated that spin system **A** is α-linked Man*p* [30,31]. The spin systems **B** and **C** (^3^*J*_1,2_ < 2 Hz) were identified as α-6dTal*p* residues [32,33]. A *J*_1,2_ coupling constant of 7 Hz and ^1^*J*_C-1,H-1_ coupling constant of 161 Hz showed that **D** was β-linked Gal*p*NAc [34]. 

**Table 3:** ^1^H and ^13^C NMR chemical shifts of the constituents of the *O*-deacetylated *O*-specific polysaccharide (OPS) of *A. bestiarum* strain K296. Spectra were recorded in D_2_O solution relative to internal acetone as reference (δ_H_ 2.225, δ_C_ 31.07).

Sugar residue	Chemical shifts (ppm)						
		H-1 C-1	H-2 C-2	H-3 C-3	H-4 C-4	H-5 C-5	H-6 C-6
→3,4)-α-d-Man*p*-(1→	**A**	5.10	4.23	4.09	3.84	3.99	3.73–3.84
		98.6	67.4	73.4	73.5	71.9	61.2
α-l-6dTal*p*-(1→	**B**	5.06	3.87	4.10	3.78	4.71	1.29
		97.6	70.9	66.3	73.4	68.2	16.3
→3)-α-l-6dTal*p*-(1→	**C**	5.01	3.90	4.03	3.87	4.15	1.24
		104.0	68.0	71.6	72.2	68.8	16.3
→3)-β-d-Gal*p*NAc-(1→	**D**	4.49	4.02	3.75	3.95	3.60	3.72–3.77
		101.6	52.5	79.9	68.4	75.8	62.3

Chemical shifts for NAc are δ_H_ 2.05 and δ_C_ 23.0/175.5 (**D**).

**Figure 4:**
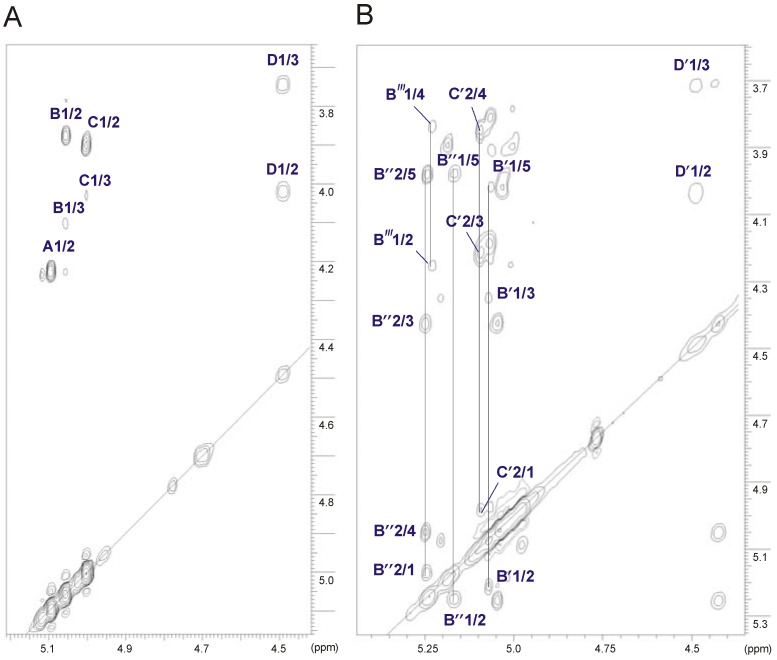
Part of a ^1^H-^1^H TOCSY spectrum of the *O*-deacetylated (**A**) and the initial OPS (**B**) of *A. bestiarum* strain K296, respectively. The maps show the spin systems of the repeating unit. Capital letters and Arabic numerals refer to atoms in the sugar residues, denoted as shown in Table 3 or in Table 4 for the *O*-deacetylated and the initial OPSs, respectively.

The stereoconfiguration of 6-deoxyhexoses was assigned according to the ^3^*J*_3,4_ and ^3^*J*_4,5_ coupling constant values, which were 3.5 Hz and less than 1 Hz, respectively, as *talo* [32,33]. In the TOCSY spectrum (Figure 4A), there were cross-peaks between H-1 and H-2,H-3, as well as correlations between H-2 and H-3,H-4 of 6dTal*p* **B** and **C**. The remaining ^1^H NMR signals of 6dTal*p* residues were established using correlations between H-6 and H-5 and between the coupled protons in the COSY and NOESY spectra. Moreover, the α-anomeric configuration of 6-deoxytaloses was also inferred from the ^1^*J*_C,H_ coupling constants (173–174 Hz) and from the *intra*-residue H-1,H-2 correlation observed in the NOESY spectrum [29,33,35]. 

The *manno*-configuration of **A** was indicated by relatively high coupling constant values of ^3^*J*_3,4_ and ^3^*J*_4,5_ (~10 Hz), contrasting with the small value of ^3^*J*_2,3_ ~3.5 Hz [30,31]. For the *manno*-spin system, cross-peaks between H-1 and H-2 and H-2 and H-3,H-4,H-5,H-6 were observed in the TOCSY spectrum. The α-configuration of Man*p* **A** was also proven by the *intra*-residue H-1,H-2 connectivity observed in the NOESY spectrum. 

The *galacto*-configuration of **D** was determined by the small ^3^*J*_3,4_ (3 Hz) and ^3^*J*_4,5_ (~1 Hz) coupling constants [34]. In the TOCSY spectrum, correlations were visible between H-1 and H-2,H-3,H-4, and the other proton signals were assigned by connectivities identified in the NOESY (strong H-3/H-5) and COSY spectra. The β-anomeric proton of Gal*p*N correlated with H-3 and H-5 in the NOESY spectrum. In addition, a *N*-acetamido sugar was confirmed by correlations of H-2 at δ 4.02 to the corresponding carbon-bearing nitrogen at δ 52.5, as revealed by the HSQC experiment. 

Low-field positions of the signals for C-3 of 6dTal*p* **C** (δ 71.6), C-3 of Gal*p*NAc **D** (δ 79.9) and C-3 and C-4 of Man*p* **A** at δ 73.4 and 73.5, as compared with the chemical shifts of the corresponding non-substituted monosaccharides, elucidated the glycosylation pattern of the sugar residues [29]. The terminal position of α-6dTal*p* **B** was confirmed by C-2-C-5 chemical shift values, which were close to those of the non-substituted α-linked sugar [35]. 

**Figure 5:**
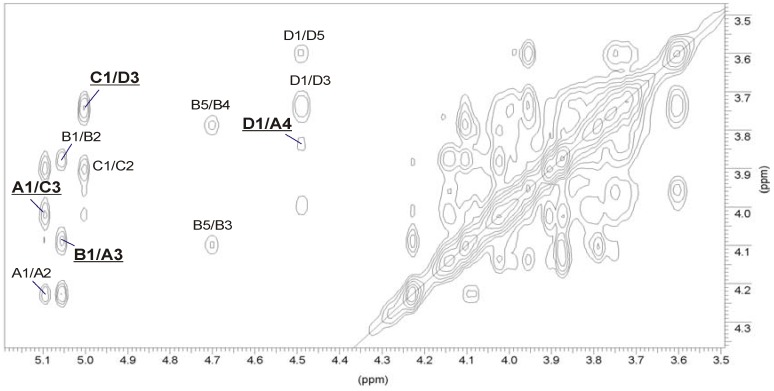
Part of a ^1^H-^1^H NOESY spectrum of the *O*-deacetylated OPS of *A. bestiarum* strain K296. The map shows NOE contacts between anomeric protons and protons at the glycosidic linkages (underlined). Some other H/H correlations are also depicted. Capital letters and Arabic numerals refer to atoms in the sugars, denoted as shown in Table 3.

The sequence of the sugar residues in the repeating unit was determined by ^1^H-^1^H NOESY and ^1^H-^13^C HMBC experiments. In the 2D NOESY spectrum of the OPS (Figure 5), the following strong NOE contacts were observed: β-Gal*p*NAc H-1 (**D**), α-Man*p* H-4 (**A**) at δ 4.49/3.84; α-Man*p* H-1 (**A**), α-6dTal*p* H-3 (**C**) at δ 5.10/4.03; α-6dTal*p* H-1 (**C**), β-Gal*p*NAc H-3 (**D**) at δ 5.01/3.75; and α-6dTal*p* H-1 (**B**), α-Man*p* H-3 (**A**) at δ 5.06/4.09. 

### 2.4. Identification of *O*-Acetylation Sites in the Initial OPS

**Figure 6:**
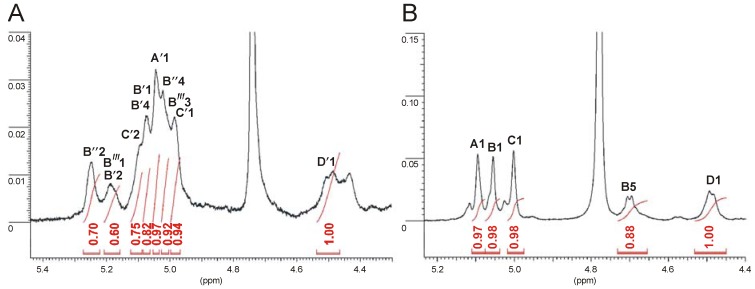
Part of ^1^H NMR spectra of the initial (**A**) and the *O*-deacetylated OPSs (**B**). Integrals are normalized for Gal*p*NAc H-1 signal.

The positions of *O-*acetyl groups attached in the native OPS were found using HSQC (Figure S2, Supplementary Information) and TOCSY (Figure 4B) experiments. All chemical shifts are summarized in Table 4. The HSQC spectrum of the initial OPS, as compared to that of the *O-*deacetylated one, demonstrated a strong downfield shift from δ_H/C_ 3.9/68.0 to δ_H/C_ 5.10/68.2 of the H-2/C-2 cross-peak of α-6dTal (**C**′), which was caused by a deshielding effect of an *O-*acetyl residue. The α-6dTal H-1/C-1 and H-3/C-3 cross-peaks shifted correspondingly from δ 5.01/104.0 and 4.03/71.6 to δ 4.98/101.3 and 4.21/71.4, respectively. Displacements of the other signals were insignificant. A comparison of integral intensities (Figure 6) in the *O-*acetylated and non-acetylated residues showed that about 80% of the 3-substituted α-6dTal (**C**′) residues were 2-*O-*acetylated. 

Besides this almost complete *O-*acetylation, several sets of signals were found, which belonged to various *O-*acetylated forms of the terminal α-6dTal (Figure 4B, Table 4). Their positions were determined on the basis of strong downfield shifts of the proton attached to a carbon atom bearing this group. Two such groups of signals were found, *i.e.*, H-2 and H-4 of the 6dTal (**B**′ and **B**″), which shifted from δ 3.87/3.78 in the deacetylated variant to δ 5.21/5.07 and δ 5.25/5.05 in 2,4-di-*O-*acetylated residues **B**′ and **B**″, respectively. *O-*Acetylation at position O-2 and O-4 of 6dTal strongly affected the shifts of H-1, H-3 and H-4 neighboring signals. The H-1/C-1 and H-3/C-3 cross-peaks shifted from δ 5.06/97.6 and 4.10/66.3 in the deacetylated variant to δ 5.08/96.5 and 4.35/64.7 and δ 5.16/96.7 and 4.42/64.6 in α-6dTal **B**′ and **B**″, respectively. The position of H-5/C-5 cross-peaks of 6dTal **B**′ and **B**″, which shifted correspondingly from δ 4.71/68.2 in the deacetylated variant to 4.03/67.4 and 3.99/67.3 in α-6dTal **B**′ and **B**″, respectively, were influenced by the *O-*acetyl group at O-4.

**Table 4:** ^1^H and ^13^C NMR chemical shifts of the initial OPS of *A. bestiarum* strain K296.

Sugar residue		Chemical shifts (ppm)					
		H-1 C-1	H-2 C-2	H-3 C-3	H-4 C-4	H-5 C-5	H-6 C-6
α-l-6dTal*p*3Ac-(1→	**B′″**	5.23	4.25	5.01	3.84	3.98	1.23
		ND	65.6	66.0	70.6	67.3	16.2
α-l-6dTal*p*2Ac4Ac-(1→	**B″**	5.16	5.25	4.42	5.05	3.99	1.23
		96.7	71.4	64.6	70.5	67.3	16.2
α-l-6dTal*p*2Ac4Ac-(1→	**B′**	5.08	5.21	4.35	5.07	4.03	1.23
		96.5	69.0	64.7	68.2	67.4	16.2
→3,4)-α-d-Man*p*-(1→	**A′**	5.06	4.19	4.00	3.75	4.02	3.76
		98.7	68.3	73.3	73.7	71.4	61.8
→3)-α-l-6dTal*p*2Ac-(1→	**C′**	4.98	5.10	4.21	3.85	4.16	1.23
		101.3	68.2	71.4	70.6	68.4	16.2
→3)-β-D-Gal*p*NAc-(1→	**D′**	4.49	4.04	3.72	3.92	3.58	3.74
		101.4	52.6	79.9	68.4	76.0	62.4

Chemical shifts for NAc are δ_H_ 2.04 and δ_C_ 23.0/175.6 (**D**′) and for OAc δ_H_ 2.15 and δ_C_ 21.2/174.2 (**C**′); δ_H_ 2.19–2.2 and δ_C_ 21.3/174.3–175.2 (**B**′); δ_H_ 2.19–2.2 and δ_C_ 21.3/174.3–175.1 (**B**″ and **B**′″); ND, not determined.

Acetylation at O-2 and O-4 was further confirmed by the correlations in the HMBC spectrum of the carboxyl carbon of the *O-*acetyl group with H-2 and H-4, which for α-6dTal **B**″ were identified at δ 175.1/5.25 and 174.3/5.05, respectively. The degree of *O-*acetylation of 6dTal **B**′ and **B**″ at O-2 and O-4 was determined by comparison of the integral intensities of proton signals in the *O-*acetylated and non-acetylated residues relative to the H-1 signal of the Gal*p*NAc residue. Integration of these signals gave 65%–70% and 70%–80% acetylation of the hydroxyl group at O-2 and O-4, respectively (Figure 6). 

In addition, a downfield displacement of the H-3/C-3 cross-peak from δ 4.10/66.3 to δ 5.01/66.0, observed in the HSQC spectrum (Figure S2), and due to the deshielding effect of the *O-*acetyl group, demonstrated acetylation of the 6-deoxytalose at position O-3 (6dTal **B**′″). The *O-*acetyl group at O-3 of **B**′″ strongly influenced the positions of H-2 and H-4 neighboring signals. The α-6dTal **B**′″ H-2/C-2 and H-4/C-4 cross-peaks shifted from δ 3.87/70.9 and 3.78/73.4 (in the *O*-deacetylated residue **B**) to δ 4.25/65.6 and 3.84/70.6, respectively. The degree of acetylation at this position was estimated at approximately 30%–40%. 

The potential level of *O*-acetylation occurring in the OPS was studied. The degree of acetylation indicated that three acetates were almost simultaneously present at positions O-2 of α-6dTal **C**′, as well as at O-2 and O-4 of the terminal α-6dTal **B**″. In addition, the mass difference, 783.27 u, which corresponded to the calculated molecular mass of the first O-unit attached to the core region, was in agreement with the NMR data and confirmed that the terminal and 3-substituted 6dTal*p* in the tetrasaccharide repeating unit are both *O*-acetylated. 

**Scheme 1:**
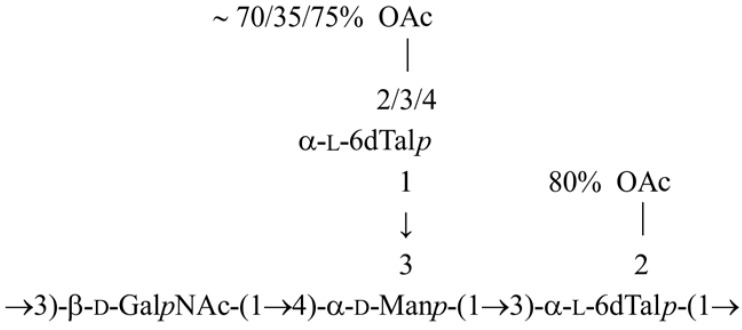
Structure of the OPS from the *A. bestiarum* strain K296.

On the basis of all the data obtained, the structure of the OPS from the *A. bestiarum* strain K296 is depicted in Scheme 1. 

Our studies have demonstrated that the OPS from *A. bestiarum* K296 O18 and *A. hydrophila* AH-3 O34 [20] are similarly composed with respect to both the sugar composition and the glycosylation pattern; however, they differ from each other in *O*-acetylation of the terminal 6dTal*p* at various positions. 

### 2.5. Immunoblotting Studies

**Figure 7:**
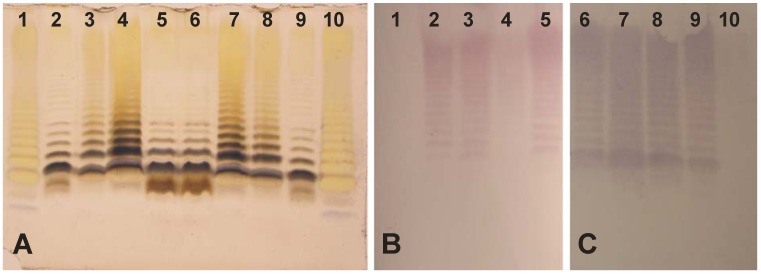
SDS-PAGE (**A**) and Western blots of the LPS preparations with anti-*A. hydrophila* AH-3 O:34 (**B**) and anti-*A. bestiarum* K296 O:18 sera (**C**), respectively. Native LPSs of *A. bestiarum* K296 (lane 3 and 8) and *A. hydrophila* AH-3 (lane 5 and 6); partially *O*-deacylated LPSs of *A. bestiarum* K296 (lane 4 and 7) and *A. hydrophila* AH-3 (lane 2 and 9). LPS of *S. enterica* sv. Typhimurium (lane 1 and 10). Approximately 3 μg of LPS was loaded per lane.

The LPS preparations from both strains were tested by Western blotting with rabbit polyclonal antisera against heat-killed bacteria. In the immunoblot, anti-*A. bestiarum* K296 O18 serum recognized mainly the slow migrating bands of the homologous and heterologous LPSs, which corresponded to high-molecular mass LPS species containing OPS (Figure 7C). A similar reaction with both LPS preparations was observed after exposition to *A. hydrophila* AH-3 O34 serum (Figure 7B), thus indicating that the cross-reactive epitopes resided on both OPSs. Only the *A. bestiarum* K296 O18 serum recognized the fast migrating bands of LPS corresponding to high molecular-mass LPS species without OPS or *R*-type LPS with one O-unit attached (*SR*-LPS phenotype), indicating a certain content of core-recognizing antibodies in the serum. Such considerable serum cross-reactivity was not surprising, as OPSs of *A. bestiarum* K296 and *A. hydrophila* AH-3 [20] strains possess similarly composed O-units. In contrast, there was a difference in the reactivity of *O*-deacylated LPS of *A. bestiarum* K296 with the *A. hydrophila* AH-3 O34 serum. The loss of antigenicity, as judged by the very weak reaction with this serum, could be accounted for by a different content of antibodies recognizing the carbohydrate and non-carbohydrate epitopes of the LPS. Moreover, structural studies revealed that the terminal 6dTal*p* in the OPS of *A. hydrophila* AH-3 was additionally 2,3- and 3,4-di-*O*-acetylated [20], thus indicating that the antibodies in the anti-*A. hydrophila* AH-3 O34 serum identified these unique LPS epitopes. Although Western blotting identified certain epitopes shared by LPSs of both strains, it could also indicate a partly different *O*-acetylation pattern of the terminal 6dTal residue in both OPSs. Alternatively, antibodies in anti-*A. bestiarum* K296 O18 serum may recognize mainly the carbohydrate epitopes. The unique structure of the *O*-antigen of *A. bestiarum* K296 and the serological data were in agreement with the classification of this bacterium in a separate serogroup. 

The structure of the *A. bestiarum* K296 OPS described herein was the third established for the species and resembled that from the OPS of *A. hydrophila* AH-3 [20]. Our data demonstrated that the irregularity in the *A. bestiarum* K296 OPS structure was associated with both non-stoichiometric *O*-acetylation and the presence of *O-*acetyl groups at different positions of the terminal 6dTal. 

Similar findings were obtained during detailed studies of the *O*-antigens from *Shigella flexneri* types 1a, 1b and 2a isolated from patients with shigellosis in Russia. The *S. flexneri* OPSs possessed a common tetrasaccharidic backbone composed of →2)-α-l-Rha*p*III-(1→2)-α-l-Rha*p*II-(1→3)-α-l-Rha*p*I-(1→3)-β-d-Glc*p*NAc-(1→ and differed from each other in their *O-*acetylation or/and glycosylation pattern, the modifications being responsible for various *S. flexneri* immunodeterminants (O-factors) [36,37]. The data obtained by Perepelov *et al.* [37] also indicated that a different degree of *O*-acetylation of Rha*p*III, which was higher at position O-3 than at O-4, suggested that the *O*-acetyl transferase for Rha*p*III was not strictly regiospecific. Alternatively, there were two *O*-acetyl transferases present, and among these, the enzyme that added the *O*-acetyl group to O-3 was more active. 

The importance of the *O-*acetylation for immunogenicity of endotoxins and polysaccharides was also shown for *Salmonella enterica* sv. Typhimurium and *Neisseria meningitidis* [36,38]. The studies of the *N. meningitidis* lipooligosaccharide (LOS) revealed that *O-*acetylation of the terminal GlcNAc in the inner core had an important role in determining the core assembly and immunotype expression and contributed to LOS structural diversity. In addition, *O-*acetylation may also influence resistance to complement-mediated killing and be important in LOS conjugate vaccine design [39]. 

## 3. Experimental Section

### 3.1. Bacterial Strain, Cultivation Conditions and Isolation of the LPS

The *Aeromonas bestiarum* strain K296 was isolated from an outbreak of motile aeromonad septicemia (MAS) in a carp farm in Poland, as previously reported [24], and was obtained from the Collection of the Microorganisms of the Department of Fish Diseases, National Veterinary Research Institute (Pulawy, Poland). The strain K296 was identified at the species level by PCR-RFLP analysis of its 16S rRNA gene sequence and classified to the serogroup O18 [21], according to the scheme of Sakazaki and Shimada [40]. 

The bacteria were cultivated in tryptic soy broth (TSB) at 28 °C for 72 h. The cells were harvested by low speed centrifugation (8000× *g*, 20 min). The recovered bacterial cell pellet was washed twice with 0.5 M saline and once more with distilled water. Bacterial cells were digested with lysozyme, RNAse and DNAse (24 h, 1 mg/g) and then with Proteinase K (36 h, 1 mg/g) in 50 mM phosphate buffer (pH 7.0) containing 5 mM MgCl_2_. The suspension was dialyzed against distilled water and freeze-dried. The digested cells were extracted three times with aq 45% phenol at 68 °C [25], and the separated layers were dialyzed against deionized water, purified by ultracentrifugation (105,000× *g*, 4 h) and freeze-dried to give LPS in a yield of 4.1% of dry bacterial cell mass. There was recovered 225 mg of LPS from the phenol phase and 352 mg from the water phase. Both LPS preparations were further analyzed. 

### 3.2. Isolation and *O*-Deacetylation of the OPS and *O*-Deacylation of the *R*-Type LPS and LPSs for Serological Studies

The OPS was obtained by mild acid hydrolysis of the *S*-type LPS (100 mg) with 2% acetic acid at 100 °C for 3 h, followed by GPC of the water soluble-portion on a column (1.8 × 80 cm) of Sephadex G-50 fine (Pharmacia, Sweden) using 1% acetic acid as an eluent and monitoring with a Knauer differential refractometer (Knauer, Berlin, Germany). The yield of the OPS fraction was 26% of the LPS mass. The sediment released by acid hydrolysis of the LPS was lyophilized to give 16.5 mg of the lipid A. 

*O*-Deacetylation was carried out by treatment of the initial OPS with 12% ammonium hydroxide at room temperature for 16 h. Then the OPS was purified by GPC on Sephadex G-50, as mentioned above.

*O*-Deacylation of the *R*-type LPS was performed in anhydrous hydrazine, as was previously described [41]. 

Partial *O*-deacylation of LPSs from *A. bestiarum* K296 and *A. hydrophila* AH-3 for serological studies was performed with 5% ammonium hydroxide (20 °C, 6 h), and after evaporation of ammonia in a stream of nitrogen, the preparations were resuspended in water and lyophilized. 

### 3.3. Chemical Analyses

For neutral and amino sugar analysis, the LPS samples and the OPS were hydrolyzed with 2 M CF_3_CO_2_H (120 °C, 2 h), *N*-acetylated, reduced with NaBD_4_ and acetylated with a 1:1 pyridine-acetic anhydride mixture (100 °C, 30 min). To release acidic sugars, LPSs and the *O*-specific polysaccharide were subjected to methanolysis (1 M HCl in methanol, 85 °C, 16 h), carboxyl reduction with NaBD_4_ in aqueous 50% methanol, hydrolysis with 2 M CF_3_CO_2_H and acetylation. The products were identified by GC-MS of alditol acetates [42] on a Hewlett-Packard HP5890A-HP5971 instrument equipped with an HP-5ms (SLB-5ms) capillary column (30 m × 0.25 mm; Supelco, St. Louis, MO, USA), applying a temperature gradient of 150 °C (5 min) to 310 °C at 5 °C min^−1^. 

The absolute configuration of monosaccharides was determined by GC of acetylated (*S*)-2-butyl glycosides using authentic sugars as standards [28]. 

Methylation of the OPS was performed by the procedure of Hakomori [43]. The permethylated OPS was subjected to hydrolysis in 2 M CF_3_CO_2_H (120 °C, 2 h), *N*-acetylation and reduction with NaBD_4_. Partially methylated alditols (PMAA) were converted into acetate derivatives and analyzed by GC-MS, as above. 

For fatty acid analysis, a sample of the lipid A (1 mg) was subjected to methanolysis in 2 M methanolic HCl (85 °C, 12 h). Methyl esters of fatty acids released were extracted with hexane and converted to their *O*-trimethylsilyl (*O*-TMS) derivatives, as described [44,45]. The methanol layer, containing the methyl glycosides, was dried and acetylated with a pyridine-acetic anhydride mixture. The fatty acids as methyl esters and *O*-TMS ethers of methyl esters, as well as acetylated methyl glycosides were analyzed by GC-MS, as above. 

### 3.4. NMR Spectroscopy

1D ^1^H NMR and 2D NMR experiments were recorded in a D_2_O solution at 32 °C using a Bruker Avance III 600 MHz spectrometer (operating frequencies 600.31 MHz for ^1^H NMR and 150.96 MHz for ^13^C NMR) and applying standard Bruker software (Bruker, TopSpin, Rheinstetten, Germany). Chemical shifts were reported relative to internal acetone as reference (δ_H_ 2.225, δ_C_ 31.07). The following homo- and hetero-nuclear correlated two-dimensional spectra were used for general assignments: ^1^H-^1^H DQF-COSY, TOCSY, NOESY, ^1^H-^13^C HSQC and ^1^H-^13^C HMBC. 1D ^13^C NMR was recorded in D_2_O at 32 °C using a Bruker DPX-360 MHz spectrometer. 

### 3.5. Mass Spectrometry Analysis

Electrospray ionization Fourier transform ion cyclotron (ESI FT-ICR) mass spectrometry was performed in negative ion mode using a hybrid Apex Qe FT-ICR MS instrument (Bruker Daltonics), equipped with a 7 Tesla actively shielded magnet and an Apollo dual ion source. Samples (~10 ng·μL^−1^) were sprayed at a flow rate of 2 μL·min^−1^. Capillary entrance voltage was set to 3.8 kV and dry gas temperature to 200 °C. For unspecific fragmentation, the DC offset (collision voltage) of the collision cell was set from 5 V to 30 V. Under these conditions, the labile linkage between the lipid A and the core oligosaccharide is cleaved [46,47], resulting in intensive Y^−^ and B^−^ fragment ions representing the lipid A and the core oligosaccharide moieties (according to the nomenclature of Domon and Costello [26]). The mass spectra were charge deconvoluted, and mass numbers given refer to the monoisotopic masses of the neutral molecules. Mass calibration was done externally by well characterized similar compounds of know structure. 

### 3.6. SDS-PAGE

LPS preparations were separated in 12.5% SDS-Tricine polyacrylamide electrophoresis gel, and bands were visualized by silver staining after oxidation with periodate, as was described [48]. 

### 3.7. Immunization Procedures and Immunoblotting

New Zealand white rabbits were acclimatized at the animal facility of the National Veterinary Research Institute (Pulawy, Poland), and all the experiments were performed according to the procedures approved by the local ethical commission. Polyclonal anti-*A. bestiarum* K296 O18 and anti-*A. hydrophila* AH-3 O34 sera were obtained by immunization of rabbits with heat-killed bacteria, as was previously described [21]. 

Western blots with rabbit antisera were performed after transferring SDS-PAGE separated LPS profiles to Immobilon P (Millipore, St. Louis, MO, USA). The primary antibodies were detected using alkaline phosphatase-conjugated goat anti-rabbit antibodies (Sigma, St. Louis, MO, USA). Blots were developed with nitroblue tetrazolium and 5-bromo-4-chloro-3-indolylphosphate toluidine (Sigma) for 5 min, as was described [48]. 

## 4. Conclusions

Species of the genus *Aeromonas* are common inhabitants of aquatic environments and have been described in connection with fish and human diseases [2,18]. The cell envelope of *Aeromonas*, as that of other Gram-negative bacteria, contains LPS, which is essential for the physical integrity and functionality of the cell membrane [49]. At present, considerable attention is being given to elucidation of the chemical structure of LPS of aquatic bacteria, which then could complete the LPS-based classification data, e.g., of strains belonging to *Aeromonas* spp. The knowledge of the defined structures of the *O*-antigens is necessary for confirmation of serospecificity of strains at the molecular level, including serological cross-reactivity between various bacterial clones and for a better understanding of the role of the *O*-antigens in bacterial pathogenesis. The variants of OPS, which represent a particular fingerprint for bacteria, might be very useful, e.g., in the identification of *Aeromonas* strains eliciting infections in farmed fish and in diagnosis of etiological agents of gastro-intestinal infections in humans. Although it was not clearly evidenced which structural determinants are the most important for virulence, it was found that some O serotypes are more frequently associated with certain infections. Studies demonstrated that *Aeromonas* strains belonging to serogroups O11, O16, O18 and O34 (Sakazaki and Shimada scheme [40]) are associated with most cases of bacteremia, implying that LPS antigens and their architecture are important in systemic disease pathogenesis [5]. 

In this work, the *S-* and *R-*type LPS preparations isolated from *A. bestiarum* K296 have been structurally characterized. It has been revealed that the rough LPS glycoforms have a hexa-acylated or tetra-acylated lipid A with amide-linked 3-hydroxy tetradecanoic fatty acids and a backbone with a conserved architecture consisting of 1,4′-bisphosphorylated-β-(1→6)-linked-d-GlcN disaccharide with an AraN residue as a non-stoichiometric substituent. The core oligosaccharide with the prevalence of heptose residues and the following composition Kdo_1_Hep_6_Hex_1_HexN_1_P_1_ has a structure shared by the core regions of the *A. bestiarum* K296 and *A. hydrophila* A-901 strains [50]. 

Herein, immunochemical examinations of the OPS were performed by chemical analyses, mass spectrometry and NMR spectroscopy. It was established that the OPS of *A. bestiarum* K296 O18 possessed a branched tetrasaccharide repeating unit composed of two 6dTal*p*, one Man*p* and one Gal*p*NAc residues. It is similar to that of the OPS of *A. hydrophila* AH-3 (serotype O34) in both the sugar composition and the glycosylation pattern; however, they differ from each other in *O*-acetylation of the terminal 6dTal*p* at various positions. Although serological studies with polyclonal rabbit sera showed that serotypes O18 and O34 shared some OPS epitopes, the cross-reactivity of the sera was affected by acetylation of the terminal 6dTal*p* residue. Such modifications of the LPS epitopes can define the serological features of particular strains and determine the classification of the bacteria. 

In future, it will be interesting to combine structural studies of LPS from different aeromonad serotypes with analyses of the location and organization of *Aeromonas* LPS gene clusters, which may shed light on their detailed chemical nature. These findings are important to characterize the *O*-antigen structures for working out new strategies for diagnosis and vaccination. 

## Supplementary Files

Supplementary File 1 Supplementary Information (PDF, 173 KB)
