# Supplementary material for: Structural and Immunochemical Studies of the Lipopolysaccharide from the Fish Pathogen, Aeromonas bestiarum Strain K296, Serotype O18
Source: Mar Drugs. 2013 Apr 17;11(4):1235–55. doi: 10.3390/md11041235 (PMC3705401; doi:10.3390/md11041235)
Supplement: Supplementary File 1 — Supplementary Information (PDF, 173 KB) [file marinedrugs-11-01235-s001.pdf]

Supplementary Information

Figure S1. ^{13}C NMR (90 MHz) (**A**) and ^{1}H NMR spectra (600 MHz) (**B**) of the *O*-deacetylated OPS of *A. bestiarum* strain K296. Capital letters and Arabic numerals refer to atoms in sugar residues denoted as shown in Table 2. A, Man*p*; B, terminal 6dTal*p*; C, 6dTal*p*; D, Gal*p*NAc; NAc, *N*-acetyl group (δ_C 23.0; δ_H 2.05); IS, acetone as internal standard (δ_C 31.07); Spectra were recorded at 32 ℃ in D_2O as a solvent.

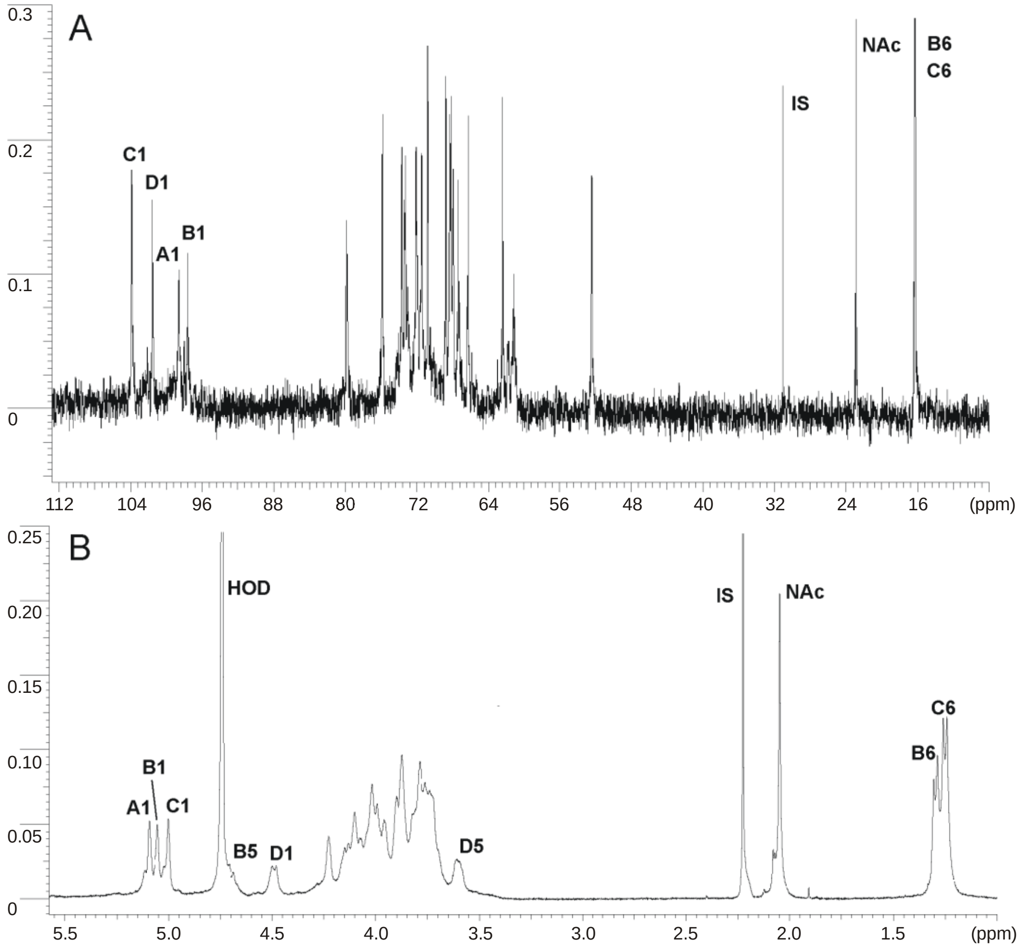

Figure S2. Part of a ^{1}H-^{13}C HSQC spectrum (600 × 150 MHz, D_2O) of the initial OPS of *A. bestiarum* strain K296. The corresponding parts of the ^{1}H and ^{13}C NMR spectra are displayed along the horizontal and vertical axis, respectively. Capital letters and Arabic numerals refer to atoms in sugar residues, denoted as shown in Table 3. A′, Man*p*; B′ and B″, 2,4-di-*O*-acetylated terminal 6dTal*p* residues; B‴, 3-*O*-acetylated terminal 6dTal*p*; C′, 2-*O*-acetylated 6dTal*p*; D′, Gal*p*NAc.

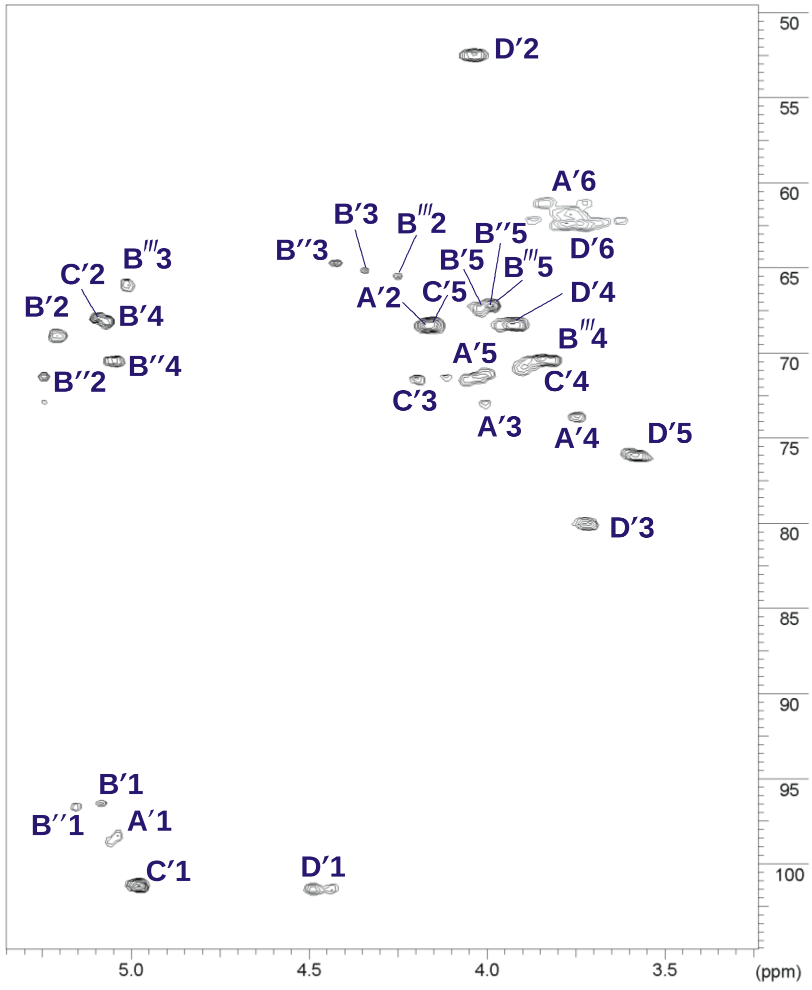

© 2013 by the authors; licensee MDPI, Basel, Switzerland. This article is an open access article distributed under the terms and conditions of the Creative Commons Attribution license (http://creativecommons.org/licenses/by/3.0/).
